# Modeling the effect of intratumoral heterogeneity of radiosensitivity on tumor response over the course of fractionated radiation therapy

**DOI:** 10.1186/s13014-019-1288-y

**Published:** 2019-05-30

**Authors:** J. C. L. Alfonso, L. Berk

**Affiliations:** 1 Department of Systems Immunology and Braunschweig Integrated Centre of Systems Biology, Helmholtz Centre for Infection Research, Braunschweig, Germany; 2 Division of Radiation Oncology, Department of Radiology, Morsani School of Medicine at the University of South Florida, Tampa, FL USA

**Keywords:** Intratumoral radiosensitivity heterogeneity, Radiation resistance, Linear-quadratic model, Fractionated radiotherapy, Accelerated repopulation

## Abstract

**Background:**

Standard radiobiology theory of radiation response assumes a uniform innate radiosensitivity of tumors. However, experimental data show that there is significant intratumoral heterogeneity of radiosensitivity. Therefore, a model with heterogeneity was developed and tested using existing experimental data to show the potential effects from the presence of an intratumoral distribution of radiosensitivity on radiation therapy response over a protracted radiation therapy treatment course.

**Methods:**

The standard radiation response curve was modified to account for a distribution of radiosensitivity, and for variations in the repopulation rates of the tumor cell subpopulations. Experimental data from the literature were incorporated to determine the boundaries of the model. The proposed model was then used to show the changes in radiosensitivity of the tumor during treatment, and the effects of fraction size, α/β ratio and variation of the repopulation rates of tumor cells.

**Results:**

In the presence of an intratumoral distribution of radiosensitivity, there is rapid selection of radiation-resistant cells over a course of fractionated radiation therapy. Standard treatment fractionation regimes result in the near-complete replacement of the initial population of sensitive cells with a population of more resistant cells. Further, as treatment progresses, the tumor becomes more resistant to further radiation treatment, making each fractional dose less efficacious. A wider initial distribution induces increased radiation resistance. Hypofractionation is more efficient in a heterogeneous tumor, with increased cell kill for biologically equivalent doses, while inducing less resistance. The model also shows that a higher growth rate in resistant cells can account for the accelerated repopulation that is seen during the clinical treatment of patients.

**Conclusions:**

Modeling of tumor cell survival with radiosensitivity heterogeneity alters the predicted tumor response, and explains the induction of radiation resistance by radiation treatment, the development of accelerated repopulation, and the potential beneficial effects of hypofractionation. Tumor response to treatment may be better predicted by assaying for the distribution of radiosensitivity, or the extreme of the radiosensitivity, rather than measuring the initial, general radiation sensitivity of the untreated tumor.

## Background

The fundamental underpinnings of radiobiology were established in 1975, when Rodney Withers proposed the four fundamental “R’s” for the response of cells to fractionated radiation therapy: Repair (the ability of the cell to repair damage from the radiation treatment), Reassortment (progression through the cell cycle, which affects sensitivity to radiation treatment), Repopulation (the rate the tumor grows during the overall treatment) and Reoxygenation (the elimination of hypoxia, which affects radiosensitivity, during treatment) [1]. In 1989, G. Gordon Steele added a fifth “R” – Radiosensitivity (the innate ability of the radiation to damage the tumor cell) [2]. In this paper, radiosensitivity refers to the cell damage directly caused by the radiation treatment under ideal conditions, and radiation sensitivity refers to the tumor cell kill from a radiation treatment incorporating all 5 “R’s” of Withers and Steele. The overall radiation sensitivity of the tumor to radiation therapy can be indicated by the SF_2_, the surviving fraction after giving a single dose of 2 Gy of radiation. It is more completely modeled in different contexts with some form of the Linear-Quadratic model of dose response [3–8].

The sensitivity of an isolated tumor cell to radiation therapy, the innate radiosensitivity, will vary due to differences of the cell’s radiosensitivity in the various parts of the cell cycle. The cells are most sensitive during G2-M phase and least sensitive in late S phase [9]. The sensitivity of a tumor cell due to its location within a tumor mass, the spatial sensitivity, varies due to processes such as hypoxia and cell-to-cell communication. A complete description of these effects was modeled by Brenner and colleagues [10]. Clinically, attempts to exploit spatial heterogeneity have been mainly focused on hypoxia-sensitizing agents, such as misonidazole and hyperbaric oxygen [11]. The variation in innate radiosensitivity has been exploited by using agents that block the tumor cell cycle from progressing into a less radiosensitive phase, such as S phase [12], or to maintain the cells in mitosis, during which cells have increased radiosensitivity [13]. In this paper, only the effects of innate radiosensitivity are being modeled. Therefore, the innate radiation sensitivity of the cells and the radiation sensitivity due to all effects, such as hypoxia and other microenvironmental factors, are identical for the purposes of this paper.

Because it can be difficult to fit clinical data to the classical linear-quadratic dose-response equation, more complex models have been developed (reviewed in [14, 15]). Recently, the “stem cell” model has been proposed to explain the apparent variation of innate radiosensitivity within a tumor [16]. In this model, the unexpected, increased resistance of a tumor to radiation therapy during fractionated therapy is modeled with two, distinct population of tumor cells. As stated by Pajonk, most if not all cancers contain a small subpopulation of cancer stem cells [16]. Rich, and others, have stated that stem cells have increased resistance to radiation therapy and may be the cause of local failure after treatment with radiotherapy [17, 18]. Yu, and others, modeled the effects of a radiation-resistant stem cell on the expected tumor response [6, 19, 20]. There is also increasing exploration of the inter-tumoral heterogeneity, that is, between patients, in innate radiation response as measured by a molecular signature. For example, Scott and colleagues presented the results of a “gene-adjusted radiation dose” (GARD) [21]. They used genetic profiling of tumors to predict radiosensitivity of several cancers and therefore their response to radiation therapy treatment. The authors showed wide heterogeneity across cancers, and that clinical outcome correlated with the GARD [21].

What has not been adequately explored is the effect of innate, intratumoral heterogeneity on tumor radiosensitivity during a standard clinical course of fractionated radiation therapy. Published preclinical data support that there is heterogeneity in the innate radiosensitivity of cancer cells in tumor masses, independent of cell cycle and microenvironmental heterogeneity. For example, Allam and colleagues studied five glioma cell lines in vitro [22], and after growth in tissue culture, each was divided into three separate specimens. They then measured the SF_2_ (surviving fraction of cells after a single 2 Gy treatment with radiation therapy) of each of these subpopulations and found an intratumoral variation in the SF_2_ of about 25%. Britten et al. performed a more complex clonal development on punch biopsies from cervical cancer [23]. They grew out 96 single cell clones from each of three specimens of squamous cervical carcinomas and then measured their radiosensitivities. The variation in the SF_2_ values was very similar to Allam’s glioma lines. Within the three original cell lines, the clones’ SF_2_ values varied from 0.240 to 0.518, 0.050 to 0.414, and 0.137 to 0.452. Thus, rather than homogeneous innate radiosensitivity in a single tumor, there is likely a range of innate sensitivities within a single tumor. The Brenner model discussed above [10], which included a term for innate radiosensitivity variation from the effect of the cell cycle, was only used to look at effects between two fractions, and discounted as unimportant any long-term effects due to variation in the innate radiosensitivity.

Therefore, a model was developed to focus on the effect of introducing intratumoral heterogeneity in innate radiosensitivity during fractionated radiation therapy. The magnitude of effects was explored by analyzing existing experimental data within the linear-quadratic equation modified with a heterogeneity factor. This model was then used to determine the effects of varying the various parameters, including the total dose, the fractional dose, number of fractions, and the rate of tumor cell repopulation.

## Methods

To model the effects of radiosensitivity heterogeneity within a tumor cell population, the standard Linear-Quadratic model for dose response [3–6] was modified with a heterogeneity component and a repopulation factor.

### The linear quadratic (LQ) model

The Linear-Quadratic (LQ) model is currently the most widely used formulation of dose-response in radiation therapy [24]. The LQ model fits in vitro cell survival experiments and incorporates the linear-quadratic behavior of observed cell survival curves [3]. Although the model was primarily derived from line-fitting [25], it is hypothesized that the linear component accounts for cell killing by DNA double strand breaks (DSBs) due to a single hit of radiation, whereas the quadratic component represents the lethal effects of two separate ionizing events that eventually cause DSBs [26, 27]. In the Linear-Quadratic equation, the surviving fraction (SF) of cells after *n* fractions of a radiation dose *d* (Gy) is given by:

1 $$ SF(d)={e}^{- nd\left(\alpha +\beta d\right)} $$

where α (Gy^− 1^) and β (Gy^− 2^) are tissue-dependent radiosensitivity parameters. It follows directly from the LQ model of Eq. (1) that the effect (*E*) of *n* equally sized fractions of dose *d* is given by *E* = *nd (α + βd)*. In turn, SF_2_, i.e. the surviving fraction of tumor cells at 2 Gy, is a defined value on this curve. This parameter is often used to compare the radiation sensitivities of tumors.

### Biologically effective dose (BED)

The biologically effective dose (BED) is a standard quantity allowing comparison of various radiation therapy fractionation schemes [28], and is dependent on the inherent biologic radiosensitivity of tissues, which is defined as the α to β ratio, α/β. This is derived from the LQ model [26], in Eq. (1), as follows:

2 $$ BED= nd\left(1+\frac{d}{\alpha /\beta}\right) $$

in which a same fractional dose (d) is delivered daily [28, 29]. This BED formalism is used to derive biologically equivalent fractionation schedules.

### Expansion of the linear-quadratic equation to reflect the effect of heterogeneous intratumoral radiosensitivity on radiotherapy response

The heterogeneous radiosensitivity of tumor cells is modeled by considering continuous distributions of intratumoral parameters α and β of Eq. (1). The innate variation in radiosensitivity of tumor cells *f*(*α*, *β*) is given by the two-dimensional Gaussian function:

3 $$ f\left(\alpha, \beta \right)={e}^{-\left(\frac{{\left(\alpha -{\alpha}_c\right)}^2}{2\ {\sigma_{\alpha}}^2}+\frac{{\left(\beta -{\beta}_c\right)}^2}{2\ {\sigma_{\beta}}^2}\right)} $$

centered at (*α*_*c*_, *β*_*c*_), and the variations in α and β distribution of tumor cells are determined by the parameters *σ*_*α*_ and *σ*_*β*_. The function *f*(*α*, *β*) is restricted to certain ranges of α and β parameters rather than infinite values by considering a cut-off of *f*(*α*, *β*) at 10^− 2^, and normalized to have integrals equal to 100% to represent the complete tumor cell population.

Combining Eqs. (1) and (3), the surviving fraction of tumor cells after a radiation dose *d* is given by *SF*(*d*) ∙ *f*(*α*, *β*). It is assumed that both the delivery of each treatment fraction and the response to radiation are instantaneous. Moreover, in this model the tumors are assumed to be homogenously irradiated. A uniform tumor cell repopulation rate per day is also assumed, i.e., a fixed percentage of the surviving cells proliferate between fractions and the percentage does not change over the course of treatment. The size of a homogenous rate of repopulation does not affect form of the radiosensitivity distributions after treatments, and only functions as a scaling factor of the absolute cell survival after treatment.

### Variation in tumor cell repopulation based on radiation resistance

The model can be further expanded to predict the effect of non-homogeneous repopulation rates among tumor cell subsets when the repopulation rate is co-varied with radiosensitivity. The intratumoral variation on repopulation *p*(*α*, *β*) is given by:

4 $$ p\left(\alpha, \beta \right)=\mu \frac{e^{\theta\ {SF}_2\left(\alpha, \beta \right)}}{\underset{\alpha, \beta }{\max }{e}^{\theta\ {SF}_2\left(\alpha, \beta \right)}} $$

where *μ* is the maximum intratumoral repopulation percentage, *θ* modulates the difference of repopulation rates between tumor cell subsets and *SF*_2_(*α*, *β*) is the intratumoral distribution of SF_2_ values with respect to α and β. Eq. (4) results in tumors with resistant cells repopulating faster compared to sensitive cells. By instead considering (1 − *SF*_2_(*α*, *β*)) in Eq. (4), then sensitive tumor cells repopulate faster than resistant cells. Combining Eqs. (3) and (4), the daily fraction of new tumor cells due to repopulation is given by *p*(*α*, *β*) ∙ *f*(*α*, *β*).

## Results

### Determination of innate radiosensitivity heterogeneity from in-vitro experiments

**Fig. 1:**
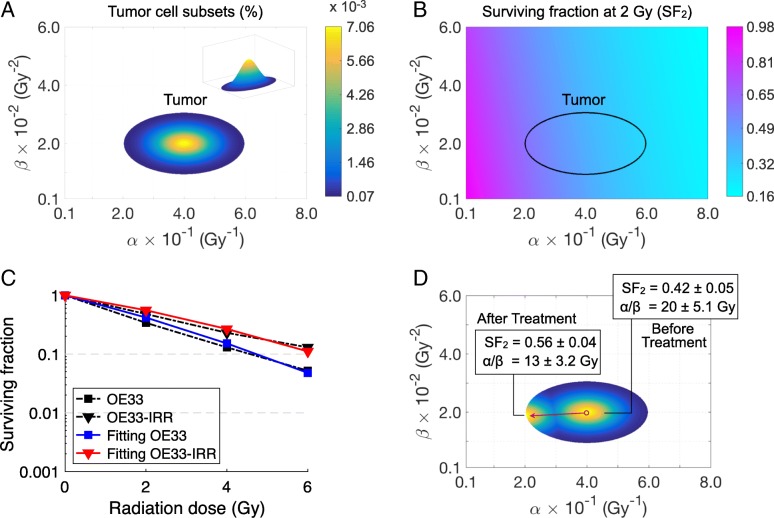
Model fitting of in vitro measurements in [30] of the change in the radiosensitivity of OE33 esophageal adenocarcinoma cell culture after exposure to fractionated radiation. **a** Representation of pre-treatment intratumoral distribution of α and β parameters. The sum of cell subset percentages is equal to 100%. **b** SF_2_ distribution with respect to α and β parameters. **c** Model fitting of tumor cell survival curves, and (**d**) the corresponding pre- and post-treatment α and β distributions. Colormap in (**d**) represents the normalized densities of each tumor cell subsets before and after treatment, where the red arrow pointing from right to left represents the evolution of α and β from pre- to post-treatment values. Pre-treatment parameters in Eq. (3) were *α*_*c*_ = 0.40 Gy^− 1^, *β*_*c*_ = 0.02 Gy^− 2^, *σ*_*α*_ = 6.5 × 10^− 2^ Gy^− 1^ and *σ*_*β*_ = 3.5 × 10^− 3^ Gy^− 2^

Quantitative in vitro measurements of the change in the radiosensitivity of tumor cell cultures after exposure to fractionated radiation therapy have been published [30, 31]. These data were used to allow realistic modeling of the tumor cell response with the addition of heterogeneity. The experimental results were modeled with the continuous distributions given by Eq. (3). The initial distribution is shown in Fig. 1a, and characterized by a distribution of innate variation in SF_2_ values as shown in Fig. 1b. The pre- and post-treatment parameters in Eq. (3) were fitted to reproduce the SF values experimentally reported. To simplify the fitting procedure, tumor cell repopulation was not considered, because the rate was not experimentally determined and the presence of homogeneous repopulation is a scaling factor that does not affect the overall results of the model (discussed below).

**Fig. 2:**
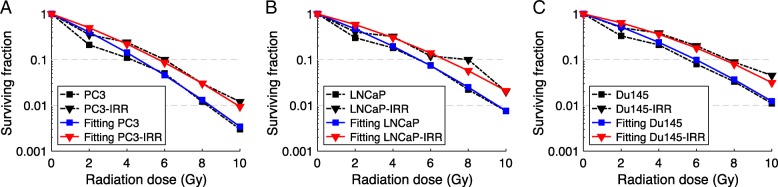
Model fitting of in vitro measurements in [31] of the change in the radiosensitivity of LNCaP, PC3, and Du145 prostate cancer cell cultures after exposure to fractionated radiation. Pre-treatment parameters in Eq. (3) were (**a**) *α*_*c*_ = 0.43 Gy^− 1^, (**b**) *α*_*c*_ = 0.35 Gy^− 1^ and (**c**) *α*_*c*_ = 0.30 Gy^− 1^ with *β*_*c*_ = 0.02 Gy^− 2^, *σ*_*α*_ = 1.0 × 10^− 1^ Gy^− 1^ and *σ*_*β*_ = 3.5 × 10^− 3^ Gy^− 2^

Figure 1c reproduces the in vitro experimental data of Lynam-Lennon and colleagues [30], in which a cell line derived from adenocarcinoma (OE33) was treated with 50 Gy in 25 daily 2 Gy fractions, and then passaged as a new, stable cell line (OE33-IRR). The surviving fractions at 2, 4 and 6 Gy of the cell line (OE33) and the cell line grown after treatment with the 50 Gy fractionated radiation therapy (OE33-IRR) were obtained. The calculated initial and post-irradiation α and β distributions are shown in Fig. 1d, where the red arrow represents the evolution from pre- to post-treatment values. Figure 2 models the data of Skvortsova et al. [31] in which three human prostate cancer cell lines (Du145, PC3, and LNCaP) were treated as per Lynam-Lennon, but with 2 Gy/day for 5 days (10 Gy total). The surviving fractions at 2, 4, 6, 8 and 10 Gy of the parental Du145, PC3, and LNCaP and radioresistant cells survived after irradiation (10 Gy) Du145-IRR, PC3-IRR and LNCaP-IRR were reported. Figure 2a-c shows that after only 5 treatments there is a permanent shift of the tumor cell population to more radioresistant clones that can be modeled with the continuous elimination of radiosensitive cells during treatment.

As shown in Figs. 1 and 2, introducing heterogeneity into the radiation resistance of the tumor successfully models the experimental data of four separate cancer cell lines. Figure 1d also highlights the inverse relationship between the pre- and post-treatment α/β ratios and the SF_2_ values. The development of a more radiation-resistant cell line during radiation treatment cannot be explained if the cells have a uniform resistance, because by definition all of the cells have the same resistance post-treatment. A dual compartment model, such as a subpopulation of radiation resistant “stem cells”, would show a rapid change of sensitivity to a second plateau of the radiosensitivity of the “stem cell”, which is not seen in the in vitro experiments.

There have been several other experimental studies on the changes induced in the radiation sensitivities of the cells due to irradiating the cells. These results are summarized in Table 1. Modeling these studies showed a rapid reduction in the α/β ratio (see Table 1) [32]. The decrease of the α/β ratio reflects the decreasing radiation sensitivity of the remaining cells, as reflected in the rising SF_2_. The rise in the SF_2_ shows that radiation therapy is less effective in killing the tumor cells after being treated previously with radiation therapy. Figures 1 and 2 also show that there is a continuous decline in the radiation sensitivity of the cells during fractionated radiation therapy, rather than being constant as assumed in the standard radiation response models.

**Table 1:** Model fitting results of in vitro measurements of the change in the radiosensitivity of cancer cell cultures after exposure to fractionated radiation. Effect of different fractionation regimens on the mean α/β ratio and SF_2_ value. For each cell line the corresponding reference of the study is provided as superindexes

Cell Line	Total Dose	Number of Fractions	α/β before treatment	α/β end of streatment	SF_2_ before treatment	SF_2_ end of treatment
PC3 ^31^	10 Gy	5	22.1 ± 7.0 Gy	16.6 ± 6.2 Gy	0.40 ± 0.09	0.50 ± 0.10
LNCaP ^31^	10 Gy	5	18.0 ± 6.5 Gy	12.5 ± 5.9 Gy	0.47 ± 0.10	0.58 ± 0.12
Du145 ^31^	10 Gy	5	15.5 ± 6.3 Gy	10.1 ± 5.5 Gy	0.52 ± 0.11	0.64 ± 0.12
A549 ^32^	16 Gy	8	10.2 ± 3.3 Gy	5.4 ± 2.7 Gy	0.44 ± 0.10	0.61 ± 0.11
H460 ^32^	26 Gy	13	3.1 ± 1.1 Gy	1.2 ± 0.8 Gy	0.46 ± 0.08	0.61 ± 0.07
OE33 ^30^	50 Gy	25	20.6 ± 5.1 Gy	13.7 ± 3.2 Gy	0.42 ± 0.05	0.56 ± 0.04

### Extension of the in vitro data to clinical treatment models

**Fig. 3:**
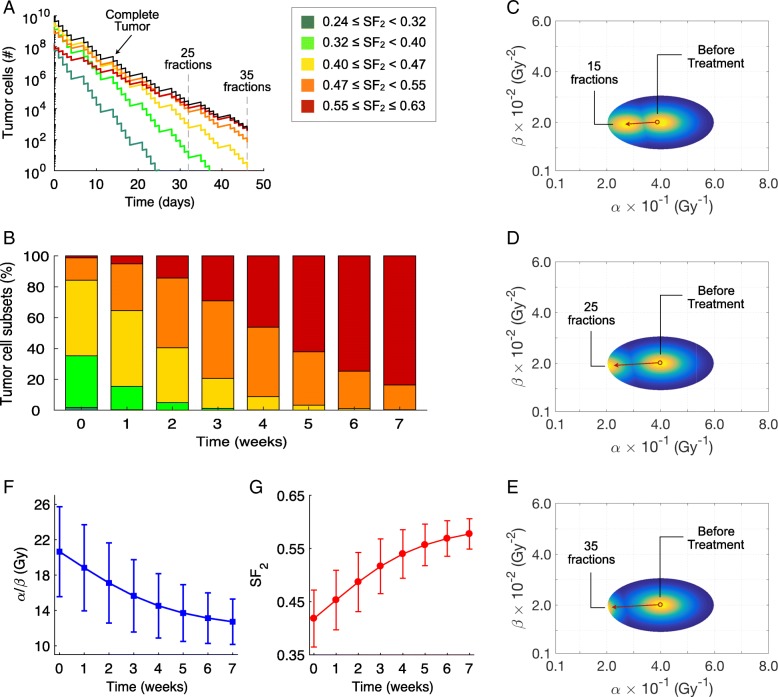
Variation of radiosensitivity during fractionated radiation therapy of a tumor as in Fig. 1. **a** Survival of tumor cell subsets characterized by different SF_2_ values in response to a standard fractionation scheme of 70 Gy in 35 daily fractions at 2.0 Gy/day with weekend interruptions. **b** Intratumoral composition of radiosensitivity for different tumor cell subsets before, during and after treatment. **c-e** Pre- and post-treatment α and β distributions after 15, 25 and 35 fractions. Colormap represents the normalized densities of tumor cell subsets before treatment and after 15, 25 and 35 fractions. The red arrows pointing from right to left represents the evolution of α and β from pre- to post-treatment values. **f-g** Variation on the mean values and standard deviations of α/β ratio and SF_2_ within the tumor during fractionated radiation therapy

The effects of innate intratumoral heterogeneity can now be modeled for extended fractionation radiation therapy as used clinically. Figure 3a shows the effect on the resistance of the tumor with pre-treatment α and β distribution derived from experimental data, as in Fig. 1, during fractionated radiation therapy of 2.0 Gy to 70 Gy in 35 fractions given over a 7-day week, with 5 days of consecutive treatment and 2 consecutive days without treatment and with an arbitrary 15% daily repopulation rate. There is a normal distribution of radiation sensitivities. As shown in Fig. 3a and b, there are initially very small populations of very sensitive and very resistant cells. It is assumed that there is initially a total of 10^10^ cells, and Fig. 3a shows the remaining number of cells (absolute) during fractionated radiation therapy of 5 treatments per week. The most-radiation-resistant populations, represented by the fraction of tumor cells with a SF_2_ ≥ 0.55, initially represent 1% of the tumor, and the most sensitive tumor cell subpopulations, represented by those cells with 0.24 ≤ SF_2_ < 0.47, represent about 85% of the tumor (Fig. 3b). By the beginning of the third week of treatment, the initially dominant, sensitive fraction becomes a minority fraction. By the end of treatment, the initially 1% clone of radiation-resistant tumor populates more than 80% of the remaining tumor and the initially dominant, sensitive tumor has been eliminated. Figure 3c-e shows the shift in the α and β distributions within the tumor after 15, 25 and 35 fractions at 2.0 Gy/day with weekend interruptions.

Figure 3 also shows the variation on α/β ratio (F) and SF_2_ (G) mean value and standard deviation within the tumor during the treatment. The α/β ratio, a measure of radiosensitivity, continuously decreases with treatment fractions while the SF_2_, a measure of radiation resistance, continuously increases. This shows that as the fractionation proceeds, each 2 Gy fraction of radiation therapy become less effective because the tumor is increasingly populated by more radioresistant cell subsets.

### The effect of the α/β ratio and distribution parameters on radiotherapy response

**Fig. 4:**
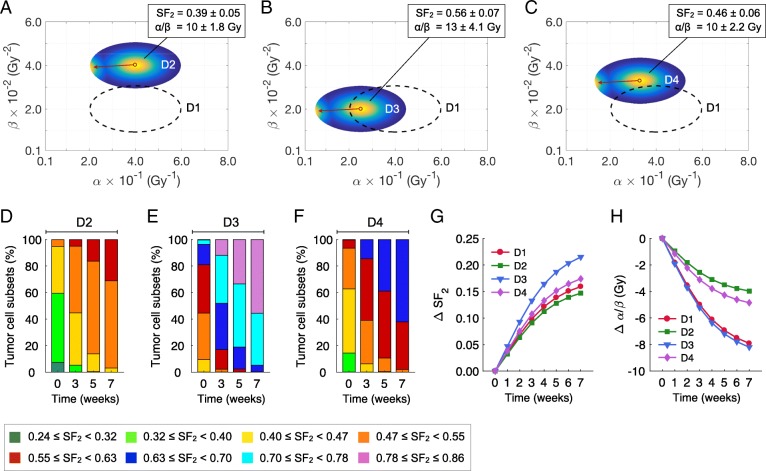
Comparison of treatment response of tumors characterized by same distribution parameters in Eq. (3) but centered at different α and β values. **a-c** Pre- and post-treatment intratumoral radiosensitivity distributions catered at different α and β combinations compared to the pre-treatment distribution in Fig. 3, dashed ellipses distribution 1 (D1), after a standard fractionation scheme of 70 Gy in 35 daily fractions at 2.0 Gy/day with weekend interruptions. Colormap represents the normalized densities of tumor cell subsets before and after treatment. The red arrows pointing from right to left represents the evolution of α and β from pre- to post-treatment values. **d-f** Intratumoral composition of radiosensitivity for different cell subsets in distribution 2 (D2) in (**a**), distribution 3 (D3) in (**b**) and distribution 4 (D4) in (**c**) before, during and after treatment. Shift in the mean values of (**g**) SF_2_ and (**h**) α/β ratio during treatment with respect to the pre-treatment values. Pre-treatment parameters in Eq. (3) were *α*_*c*_ = 0.40 Gy^− 1^ and *β*_*c*_ = 0.04 Gy^− 2^ (D2 in **a**), *α*_*c*_ = 0.25 Gy^− 1^ and *β*_*c*_ = 0.02 Gy^− 2^ (D3 in **b**), and *α*_*c*_ = 0.33 Gy^− 1^ and *β*_*c*_ = 0.033 Gy^− 2^ (D4 in **c**) with *σ*_*α*_ = 6.5 × 10^− 2^ Gy^− 1^ and *σ*_*β*_ = 3.5 × 10^− 3^ Gy^− 2^

Clinical measurements from the treatment of patients with cancers show that the α/β ratios of intact, human tumors have a wide range of values [33]. The most common approximation is to use an α/β ratio for a malignant tumor of 10 Gy. However, the in vitro cell lines often have higher α/β ratios, closer to 20 (see Figs. 1 and 2) [30, 31, 34]. The relative effect of the development of radiation resistance during treatment is not strongly dependent on the initial radiosensitivity parameters. This is shown in Fig. 4, which compares the effects of fractionated radiotherapy (70 Gy in 35 daily fractions at 2.0 Gy/day with weekend interruptions) on the resistance of tumors with a pre-treatment α and β distribution width as in Fig. 3, but centered at different α and β combinations. Distribution 1 (D1) is the original α/β ratio modeled in Fig. 3 and is represented by the dashed ellipses in Fig. 4a-c. Distribution 2 (D2) in Fig. 4a is centered at a higher β but the same α, and thus a lower α/β ratio. Distribution 3 (D3) in Fig. 4b is centered at the same β but a lower α, and thus has a lower α/β ratio. Distribution 4 (D4) in Fig. 4c is centered at both a lower α and a higher β, but at the same ratio as Distribution 2, allowing exploration of the effects of the α and β at the fixed ratio.

**Fig. 5:**
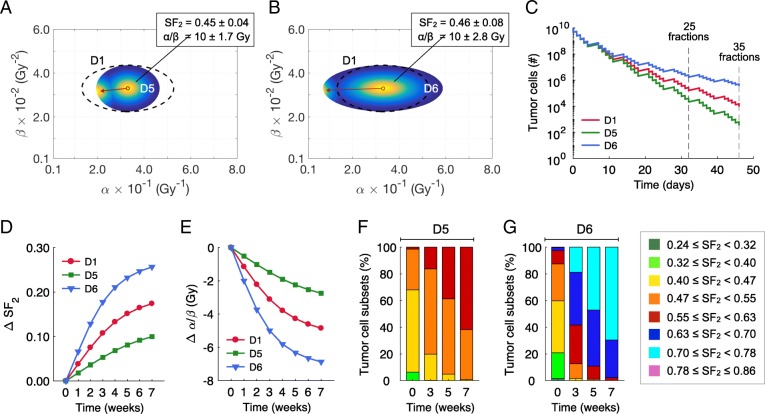
Comparison of treatment response of tumors characterized by different distribution widths in Eq. (3) and centered at same α and β parameters. **a-b** Pre- and post-treatment α and β distributions of different radiosensitivity heterogeneities (widths) compared to the pre-treatment distribution 4 (D4) in Fig. 4c (dashed ellipses) after a standard fractionation scheme of 70 Gy in 35 daily fractions at 2.0 Gy/day with weekend interruptions. Colormap represents the normalized densities of tumor cell subsets before and after treatment. The red arrows pointing from right to left represents the evolution of α and β from pre- to post-treatment values. **c** Tumor cell survival in response to fractionated radiotherapy. Shift in the mean values of (**d**) SF_2_ and (**e**) α/β ratio during treatment with respect to the pre-treatment values. **f-g** Intratumoral composition of radiosensitivity for different cell subsets in distribution 5 (D5) in (**a**) and distribution 6 (D6) in (**b**) before, during and after treatment. Pre-treatment parameters in Eq. (3) were *σ*_*α*_ = 4.5 × 10^− 2^ Gy^− 1^ (D5 in **a**) and *σ*_*α*_ = 8.5 × 10^− 2^ Gy^− 1^ (D6 in **b**) with *σ*_*β*_ = 3.5 × 10^− 3^ Gy^− 2^, *α*_*c*_ = 0.33 Gy^− 1^ and *β*_*c*_ = 0.033 Gy^− 2^

In all simulated cases, there is a rapid shift of the tumor cell population to more resistant clones, with higher SF_2_ values and lower α/β ratios during treatment. However, Fig. 4d-f shows that this shift is much more pronounced when α is lowered, rather than β raised, reflecting that at 2 Gy doses the α part of the dose response curve is dominant. Thus, induction of radiosensitivity is due primarily to the shift in α, with β remaining relatively stable (see Figs. 1, 3, 4 and 5). Figure 4g-h shows that at all modeled α/β ratios there is no evidence of a plateau in the radiation sensitivity, and that tumors become more radiation resistant as the treatment proceeds.

The effect of the variation on the width of the intratumoral radiosensitivity heterogeneity, given by different α and β distributions around an α/β ratio of 10 Gy, on the effects of fractionated radiation therapy (70 Gy in 35 daily fractions at 2.0 Gy/day with weekend interruptions) was also explored. In Fig. 5, the α and β distribution in Fig. 4c is shown with the dashed ellipses (distribution 1, D1). Figure 5a models a tighter distribution (D5), and Fig. 5b models a wider distribution (D6). Wide distributions result in more heterogeneous tumors after treatment with more radioresistant tumor cells, as compared with tighter distributions, which are dominated by relatively more sensitive cell subsets. Increased tumor heterogeneity results in a smaller reduction in tumor cell population (tumor cell kill) during treatment (Fig. 5c) due to the resistant clones, and more pronounced shifts in the SF_2_ value (Fig. 5d) and α/β ratio (Fig. 5e) by the end of treatment. At the end of 7 weeks of treatment a tumor with a tight distribution is still primarily made up of moderately radiosensitive cells (Fig. 5f), whereas resistant cells populate the tumor with a wide distribution (Fig. 5g).

### The effect of hypofractionation on radiosensitivity

**Fig. 6:**
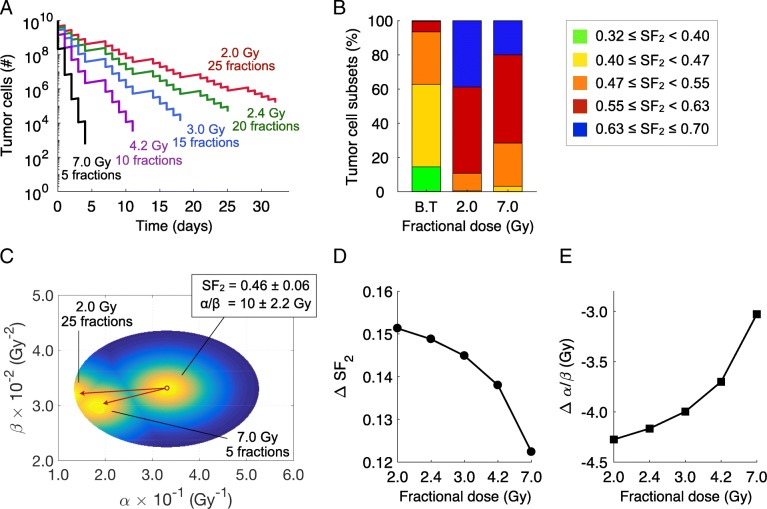
Variation of intratumoral radiosensitivity during BED equivalent fractionated radiation therapy. **a** Survival of a tumor to different BED-equivalent radiation therapy fractionation schemes at 2.0Gy/day, 2.4Gy/day, 3.0Gy/day, 4.2Gy/day and 7.0Gy/day in 25, 20, 15, 10 and 5 fractions with 5 consecutive treatments per 7-day week. **b** Intratumoral composition of radiosensitivity for different tumor cell subsets before and after treatments at 2.0Gy/day and 7.0Gy/day in 25 and 5 fractions with 5 consecutive treatments per 7-day week. **c** Intratumoral distribution of radiosensitivity parameters α and β before and after treatments. The red arrows pointing from right to left represents the evolution of α and β from pre- to post-treatment values. Shift in the mean values of (**d**) SF_2_ and (**e**) α/β ratio within the tumor at the end of BED-equivalent fractionation schemes. Pre-treatment parameters in Eq. (3) were *α*_*c*_ = 0.33 Gy^− 1^ and *β*_*c*_ = 0.033 Gy^− 2^ with *σ*_*α*_ = 6.5 × 10^− 2^ Gy^− 1^ and *σ*_*β*_ = 3.5 × 10^− 3^ Gy^− 2^ corresponding to the distribution 4 (D4) in Fig. 4c

Figure 6 shows the effect of daily fraction size on the cell death and post-treatment intratumoral radiosensitivity for a α/β ratio of 10 Gy and a BED of 60 Gy. Standard fractionation of 50 Gy in 25 daily fractions at 2.0 Gy/day with weekend interruptions is compared with four increased daily fraction doses with the same BED, and, by definition, the same expected total cell death. These are all delivered five days per week. These treatments are: (*i*) 20 fractions at 2.4 Gy/day, (*ii*) 15 fractions at 3.0 Gy/day, (*iii*) 10 fractions at 4.2 Gy/day, and (*iv*) 5 fractions at 7.0 Gy/day. 50 Gy, rather than 70 Gy, was used as the standard treatment protocol to decrease the spread of survival between the different treatment arms and allow better graphical comparison of the results. In the model, increasing the daily fraction size decreases the overall survival of the tumor cells at the end of treatment, despite being formally BED equivalent (Fig. 6a). In Fig. 6b, “B.T” corresponds to the distribution of radiosensitive cells of the untreated tumor, i.e. before treatment. Figure 6b shows that the lower daily dose fractionation regimens result in more radiation-resistant tumors. Figure 6c compares the corresponding shifts in the α and β distribution within the tumor after 25 fractions of 2 Gy/day and 5 fractions of 7.0 Gy/day. Figure 6d-e, which plot the change in SF_2_ values and α/β ratios after treatment, shows that lower daily doses result in increased shifts in mean *α*/*β* ratio and SF_2_ value. This suggests that there is an increasing efficiency of tumor kill with increasing dose per fraction size at *theoretically* iso-equivalent BED total doses due to heterogeneity of radiosensitivity.

### The effect of variation in tumor cell repopulation on treatment response

**Fig. 7:**
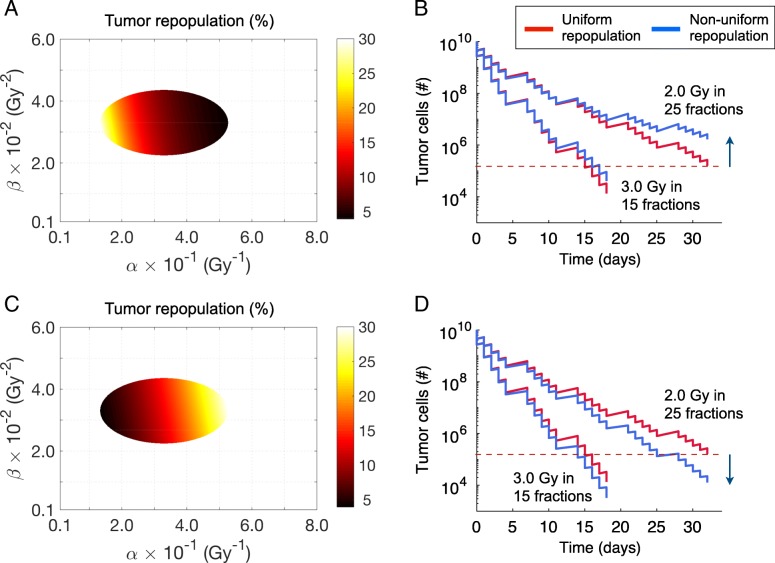
Radiotherapy response of tumors with more rapidly and slowly growing resistant cells. **a**, **c** Non-uniform distribution of daily repopulation rates, (**a**, **b**) rapidly and (**c**, **d**) slowly growing resistant cells, within a tumor as in Fig. 4c. Parameters in Eq. (4) were *μ* = 0.3 and *θ* = 5.5. **b**, **d** Tumor cell survival to BED-equivalent fractionations at 2.0 Gy/day in 25 fractions and 3.0 Gy/day in 15 fractions both with weekend interruptions. Results were obtained for a uniform repopulation of 15% and the non-uniform repopulation distributions as in (**a**, **c**)

The previous calculations assumed a uniform repopulation rate among the tumor cells. However, it is also possible that the resistant cells could grow faster or slower than the sensitive cells. Figure 7 compares the effect of uniform and non-uniform repopulation rates among the resistant cells. If there is a faster repopulation rate among the more resistant cells then the survival curve is above the standard (uniform) curve (Fig. 7a-b). If there is a slower repopulation rate among the resistant cells, the survival curve will go under, rather than over, the standard curves (Fig. 7c-d). The effect of a different repopulation rate among resistant cells is more pronounced for prolonged fractionation than it is for shorter courses of treatment. Shortening the treatment time with hypofractionation causes a marked reduction in the population of resistant cells at the end of treatment when compared with standard fractionation. If the resistant cells repopulate faster than the sensitive cells, then the effect is equivalent to the accelerated repopulation that is seen in clinical treatment, in that as the treatment progresses the tumor growth rate increases due to the increasing percentage of rapidly growing, radioresistant cells.

## Discussion

Cancers are genetically diverse, not only between patients but also within an individual patient [35–39]. This perhaps obvious fact has not been evaluated in previous models of radiation response. Pre-clinical data, such as that modeled in Figs. 1, 2 and 3, support the model that radiation therapy rapidly selects out the more radiation-resistant clones of tumor over the dominant, more sensitive cells initially present. Along with the experimental results discussed previously, other experiments report similar findings. McDermott and colleagues exposed a prostate cancer cell with 60 Gy of radiation therapy in 30 treatments (RR cells) and compared the radiation sensitivity of the resulting cell line with the untreated wild type cell line (WT) [40]. They found increased post-radiation survival, decreased baseline apoptotic rates, and increased DNA repair capacity of the RR cells as compared to the WT cells. This was also shown in cell lines from esophageal cancer [41], and another cervical cancer line [42]. These results can be successfully modeled within standard linear-quadratic mechanics by assuming a distribution of radiosensitivity within a single tumor. This expansion of the fifth R (Radiosensitivity) with a distribution of innate radiosensitivity provides a basis for the induction of radiation resistance by the treatment with radiation therapy during treatment and for the clinical phenomenon of accelerated repopulation during treatment. This induction of radiosensitivity is due primarily to the shift in α, with β remaining relatively stable. This is congruent with the increased repair capacity found in the experimental studies.

An important focus of radiation research is to predict when and what type of radiation treatments will be effective. For example, one area of current research is using molecular analysis of tumors to predict radiosensitivity. A similar approach was explored in the 1990’s, using measured SF_2_ values, but this approach was ultimately abandoned due to a lack of sufficient correlation with clinical outcome [43]. The results of this heterogeneity model suggest that the local-control-limiting, resistant cells may be a very small population within a large population of sensitive cells. Therefore, measurements made on the pre-treatment tumor may not be able to detect the most important subpopulation. What may be more effective for predicting local response is to determine the distribution of the radiosensitivities in the tumor. Approaches that could overcome this limitation include: making several measurements early in the treatment and monitoring the shift in radiosensitivity, using a technique that detects the small, most radiation-resistant clones in the tumor, or directly measuring the initial width of the distribution of the tumor radiosensitivity.

Clinical radiation therapy has a limited range of variables to change to try to improve the outcome of treatment. The primary variables are the total dose, the daily dose and the length of treatment. This model suggests that increasing the total dose to tumors that are not cured with standard doses of radiation therapy will not be effective because the remaining cells are the most radiation-resistant cells. That is, each subsequent fraction of radiation therapy is increasingly inefficient in curing the cancer because of the increase in radiation resistance with each treatment fraction. The modeling of Fig. 6 shows that increasing the daily dose and decreasing the total time of the treatments result in higher tumor kill without the development of a predominant radiation-resistant clone. The model indicates that hypofractionation will induce less radioresistance for the same calculated BED. This was shown to be true in vitro for two cell lines by Zhang et al. using dose-response curves [32]. They also showed by using flow cytometry that longer fractionation induced more “stem cells”, which by this model are the more radioresistant cells. The limitation of hypofractionation is the concomitant increase in damage to the normal tissue, and a decreased therapeutic ratio, in as much as larger fractions are also more efficient in killing the normal cells.

The model also offers an explanation of the clinically seen problem of accelerated repopulation during extended treatment (Fig. 7). The experimental results of McDermott, mentioned above [40], as well as Kuwuhara [41], Lynam-Lennon [30] and Skvortsova [31], all report in vitro data showing that the radiation-induced clones are both more radiation resistant and have a faster growth rate than the original cell line. Thus, as shown in Fig. 7, the selection of the rapidly growing, resistant fraction of the tumor during extended fractionation supplies a simple explanation of the clinically identified phenomenon of accelerated repopulation during treatment of cancers, and the advantage of shorter treatment times. The model also shows that decreasing the time of the treatment minimizes this affect, also as seen in clinical studies.

## Conclusions

The presence of a distribution of innate radiosensitivity within a single tumor can explain many aspects of clinical radiobiology, including the development of radiation resistance during radiation therapy, accelerated repopulation during treatment, and the lack of a significant improvement in cure rates with higher doses over standard doses for many clinically-treated cancers. It also predicts that assays of radiation resistance based on the tumor prior to treatment may not be the most sensitive predictors of radiation response because they may not measure the control-limiting, resistant cells. Alternative approaches, such as measuring the distribution of the tumor resistances, or using a technique to select out the radiation resistant cells prior to the assay, may be more adequate.

## Abbreviations

BED: Biologically effective dose
DSBs: Double strand breaks
GARD: Gene-adjusted radiation dose
LQ model: Linear-quadratic model
SF: Surviving fraction
WT: Wild type
